# Polyphenol-Capped Biogenic Synthesis of Noble Metallic Silver Nanoparticles for Antifungal Activity against *Candida auris*

**DOI:** 10.3390/jof8060639

**Published:** 2022-06-16

**Authors:** Maqsood Ahmad Malik, Maha G. Batterjee, Majid Rasool Kamli, Khalid Ahmed Alzahrani, Ekram Y. Danish, Arshid Nabi

**Affiliations:** 1 Chemistry Department, Faculty of Science, King Abdulaziz University, P.O. Box 80203, Jeddah 21589, Saudi Arabia; mbatterjee@kau.ed.sa (M.G.B.); kaalzahrani1@kau.edu.sa (K.A.A.); eydanish@kau.edu.sa (E.Y.D.); 2 Department of Biological Sciences, Faculty of Sciences, King Abdulaziz University, P.O. Box 80203, Jeddah 21589, Saudi Arabia; mkamli@kau.edu.sa; 3 Center of Excellence in Bionanoscience Research, King Abdulaziz University, Jeddah 21589, Saudi Arabia; 4 Department of Chemistry, University of Malaya, Kuala Lumpur 50603, Malaysia; arshidpharmachem@gmail.com

**Keywords:** green synthesis, polyphenols, cell cycle, *Candida auris*

## Abstract

In terms of reduced toxicity, the biologically inspired green synthesis of nanoparticles has emerged as a promising alternative to chemically fabricated nanoparticles. The use of a highly stable, biocompatible, and environmentally friendly aqueous extract of *Cynara cardunculus* as a reducing and capping agent in this study demonstrated the possibility of green manufacturing of silver nanoparticles (CC-AgNPs). UV–visible spectroscopy validated the development of CC-AgNPs, indicating the surface plasmon resonance (SPR) λ_max_ band at 438 nm. The band gap of CC-AgNPs was found to be 2.26 eV. SEM and TEM analysis examined the surface morphology of CC-AgNPs, and micrographs revealed that the nanoparticles were spherical. The crystallinity, crystallite size, and phase purity of as-prepared nanoparticles were confirmed using XRD analysis, and it was confirmed that the CC-AgNPs were a face-centered cubic (fcc) crystalline-structured material. Furthermore, the role of active functional groups involved in the reduction and surface capping of CC-AgNPs was revealed using the Fourier transform infrared (FTIR) spectroscopic technique. CC-AgNPs were mostly spherical and monodispersed, with an average size of 26.89 nm, and were shown to be stable for a longer period without any noticeable change at room temperature. Further, we checked the antifungal mechanism of CC-AgNPs against *C. auris* MRL6057. The minimum inhibitory concentrations (MIC) and minimum fungicidal concentrations (MFC) were 50.0 µg/mL and 100.0 µg/mL respectively. The cell count and viability assay confirmed the fungicidal potential of CC-AgNPs. Further, the analysis showed that CC-AgNPs could induce apoptosis and G2/M phase cell cycle arrest in *C. auris* MRL6057. Our results also suggest that the CC-AgNPs were responsible for the induction of mitochondrial toxicity. TUNEL assay results revealed that higher concentrations of CC-AgNPs could cause DNA fragmentation. Therefore, the present study suggested that CC-AgNPs hold the capacity for antifungal drug development against *C. auris* infections.

## 1. Introduction

Green nanotechnology is a fast-emerging science with potential applications in the pharmaceutical, healthcare, biomedical, and drug delivery fields [1,2,3]. It was reported that a variety of metallic nanoparticles, including gold and silver nanomaterials, are being developed for use in a wide range of scientific applications [4]. Because of their excellent antioxidant and antibacterial capabilities, plasmonic silver nanoparticles (AgNPs) have recently received a lot of attention [5,6]. The surface plasmon resonance (SPR) of metal nanoparticles makes them interesting because of their applications in photocatalysis, sensors, biodevices, drug storage and loading, antimicrobial activity, and spectroscopic applications [7,8,9,10,11]. The SPR of the metal nanoparticles depends on the shape, size, and surrounding dielectric medium, as SPR is the resonant oscillation of conduction electrons under appropriate light illumination [12,13,14]. Silver nanoparticles are well-known noble metallic materials with strong antimicrobial and photocatalytic properties because of their high sensitivity, chemical stability, and better light absorption and optical properties [15,16,17].

The synthesis of metal nanoparticles involves various physical and chemical routes, which are quite expensive, require high energy, and have various toxicity issues associated with these approaches [18], therefore new cost-efficient, non-toxic, and eco-friendly synthesis techniques were adopted [19]. Bioactive substances, such as plant materials and microbes, and biowastes, such as vegetable waste, fruit peel trash, eggshell, and agricultural waste; can be used to synthesize different metal nanoparticles [20]. The restrictions of synthetic approaches are overwhelmed using green chemistry methods, which are economical and require less time to synthesize nanoparticles. Hence, many researchers performed the synthesis of nanoparticles via a green chemistry approach [21,22,23]. The metabolites present in the plant extract play a significant role in the reduction, nucleation, growth, stability, and capping of the silver nanoparticles [24]. The reducing capacity of plant extracts depends on water-soluble phenolic compounds, which have a key function in the reduction of Ag ions [24]. The method used to prepare metallic nanoparticles, nature of the solvent, mixing ratio, concentration, pH, temperature of the reaction mixture, and strength of the reducing agent are all key factors that influence the size, morphology, and stability of the nanoparticles [25,26,27]. Furthermore, Kim et al. deduced the concentration-dependent inhibitory cytotoxicity against Escherichia coli and Staphylococcus aureus using silver nanoparticles within a range of 13.5 nm [28]. Pauksch et al. studied cell proliferation, viability, and bone-forming cells upon incubation with AgNPs over time and in a dose-dependent manner [29]. Further, emphasis on AgNPs in biomaterials may lead to decreased cytotoxicity due to the possible reduced chance of AgNP cellular uptake; meanwhile, a window may open for future AgNP clinical and pharmaceutical applications in real-time medicinal practice. Along similar lines, the biocompatibility of biogenic AgNPs was investigated by P. Kumar Panda et al. in zebrafish embryos. However, both computational and experimental analysis was utilized in a concentration-dependent manner, the AgNPs enhanced oxidative stress accumulation and internalization depending on an intrinsic atomic interaction with the proteins, including sod1, tp53, and apoa1-mttp. In addition, it was ascertained that the biogenic AgNPs developed from silver grass were significantly biocompatible and eco-compatible and could be used for biomedical and ecological applications [30]. Silver nanoparticles, those developed via biogenic essence are proven to be biocompatible, such as oligodynamic characteristics of such biogenic NPs have been explored for thousands of years ago. In particular, cups made up of silver were used as a therapeutic agent in the Roman Empire [31]. It is worth mentioning that based on the inherent microbial inhibition against fungi and bacteria on the surface of AgNPs, this makes them a comparatively efficient antimicrobial candidate relative to other biogenic metal nanoparticles [32,33,34]. The biocompatibility of AgNPs was further ascertained after the continuous release of small amounts of silver ions from the surface of AgNPs, which was responsible for the inhibition of bacterial growth on the surface of nanoparticles, as well as on the metal surface. In real life medical applications, AgNPs are being used in medical operations, including impregnated catheters and in wound dressings [35]. Moreover, the AgNPs are being used as highly antibacterial agents nowadays and show potent efficacy as antimicrobial agents at concentrations ≤10 µg/g and retain an efficient potency against biofilm formation, as reported in previous studies [28,36,37,38,39,40]. Keeping the biogenic and biocompatible yield of polyphenol-capped silver nanoparticles, along with their inherent antimicrobial properties, in mind, here we aimed to investigate the antifungal activities against *C. auris* strains.

Silver nanoparticles synthesized using green chemistry approaches show antioxidant [41], antibacterial, and anti-inflammatory properties [42]. Recently, bloodstream infections caused by *Candida auris* have been spreading and widely reported in different parts of the globe [43]. This species of Candida was initially reported in 2009 in Japan [44] as an evolving multidrug-resistant (MDR) yeast pathogen and is mainly responsible for septicemia, resulting in a high rate of mortality. The spread of *C. auris* is identified as a risk in healthcare units and leads to outbreaks. Additionally, unlike other species of *Candida*, this pathogen can persist and flourish for a long time on both dry and moist surfaces in clinics and hospitals [45,46]. The MDR property of *C. auris* was described [47] and the scenario becomes further complicated by the formation of biofilms [48] and active efflux pumps. Therefore, there is a need to look for new and efficient antifungal strategies to combat this evolving yeast pathogen and prevent nosocomial outbreaks. Considering the importance of silver nanoparticles, the present work deals with the *Cynara cardunculus* extract assisted preparation of silver nanoparticles using a simple, nontoxic, and economical approach. The structural properties of CC-AgNPs were investigated using different spectroscopic and microscopic techniques to determine the surface morphology, elemental composition, crystallinity, and optical properties. Further, the present study aimed to investigate the antifungal activities of CC-AgNPs against *C. auris* strains.

## 2. Materials and Methodology

### 2.1. Materials

*Cynara cardunculus*, commonly known as artichoke, was collected from a local market in Jeddah, Saudi Arabia. Silver nitrate (AgNO_3_, ≥99.0%) and ethanol (CH_3_CH_2_OH, 95.0%) were purchased from Sigma–Aldrich, St. Louis, MO, USA. All the chemicals used in this study were of analytical grade and were used without additional treatment. Highly pure double-distilled water (DDW) was utilized for the preparation of silver precursor and *Cynara cardunculus* solution.

### 2.2. Preparation of Cynara cardunculus Extract

The collected *Cynara cardunculus* were washed several times with distilled water, dried until the moisture was completely removed, and then ground into a fine powder. The *Cynara cardunculus* powder (10 g) was dispersed in an Erlenmeyer flask containing 250 mL distilled water and further heated at 80 °C for 60 min to achieve the completed extraction of biomolecules. The unfiltered solution was kept at room temperature for 12 h, and after that, the resulting extract was filtered through Whatman filter paper No. 1 using vacuum filtration apparatus. The filtered aqueous solution of *Cynara cardunculus was* stored in a refrigerator at 4 °C for further experimental use. It is recommended that fresh *Cynara cardunculus* extract (no more than 5 days after extraction) is used for synthesizing CC-AgNPs.

### 2.3. Preparation and Physicochemical Characterization of CC-AgNPs

The preparation of the silver nanoparticles was initiated by optimizing the amount of *Cynara cardunculus* extract required for the synthesis of CC-AgNPs. After several optimizing experiments, 14 mL of *Cynara cardunculus* extract was added to 20 mL of 1.4 × 10^−4^ M silver nitrate solution under continuous stirring using a magnetic stirrer. The color of the reaction mixture changed after just 5 min of reaction time and was analyzed using a double-beam Thermo Scientific Evolution 300 UV–visible spectrophotometer. The *Cynara*-*cardunculus*-mediated CC-AgNPs were purified, and the precipitated pellets were collected by using a BIOBASE centrifuge at a centrifugation speed of 5000 rpm for 20 min. The acquired pellets were then dispersed in distilled water and successively washed several times to completely remove the unbound compounds from the surface of the CC-AgNPs. Furthermore, the obtained material was subsequently dried at 90 °C for 5 h and then calcined at 500 °C for 3 h in a muffle furnace to remove all surface impurities and increase the crystallinity.

The successful reduction of the silver metal ions using *Cynara cardunculus* extract was initially validated by recording the absorbance of the reaction mixture in the wavelength range of 200–800 nm using a double-beam Thermo Scientific Evolution 300 UV–visible spectrophotometer (Thermo Fisher Scientific, Waltham, MA, USA). All spectra were recorded at room temperature, in a quartz cuvette cell (path length 1 cm). A powder X-ray diffractometer (XRD) (D8 Advance, Bruker, Karlsruhe, Germany) set to 40 kV and 40 mA with 1.54 Å CuKα radiation was used to acquire the XRD pattern of the as-prepared CC-AgNPs in the scan range of 20–80 θ. Fourier transform infrared spectroscopy (FTIR) analysis of CC-AgNPs was performed on a Bruker ALPHA II FT-IR (Bruker Optics GmbH & Co., Rosenheim, Germany) spectrometer to assess the possible involvement of the functional groups in the *Cynara cardunculus* extract in the reduction and stabilization/capping of the CC-AgNPs. Transmission electron microscopy (TEM) (JOEL, JEM-2100F, Tokyo, Japan; accelerating voltage of 200 kV) measurements were performed to analyze the morphology and the particle size distribution of the CC-AgNPs. Scanning electron microscopy (SEM) (ZEISS-SEM, Oberkochen, Germany) equipped with an energy dispersive spectroscopy (EDS) was used to investigate the surface morphology and the elemental composition of the CC-AgNPs. Malvern Zetasizer (Malvern Panalytical Ltd., Enigma Business Park, Malvern, UK) examined the zeta potential and the particle size distribution of the CC-AgNPs. The thermal stability of the as-prepared CC-AgNPs was analyzed using thermogravimetric analysis (TGA) in the temperature range of 30–800 °C under a N2 atmosphere with a heating rate of 10 °C/min using a Perkin-Elmer Pyris Diamond thermogravimetric analyzer (PerkinElmer LAS (UK)Ltd., Llantrisant, UK).

### 2.4. Antifungal Activity of CC-AgNPs

In the present study, the *C. auris* clinical strain MRL6057 was used. The strain was obtained from the National Institute of Communicable Diseases (NICD), South Africa, and preserved in the department as a glycerol stock. The antifungal action of CC-AgNPs was evaluated against *C. auris* MRL6057 by using a broth microdilution assay recommended in the standard M27 document (fourth ed.) [49]. The concentrations used for the test NPs and positive control/amphotericin B (AmB; Sigma-Aldrich, St. Louis, MO, USA) were 200–0.19 µg/mL and 16–0.031 µg/mL, respectively. Before reading the MIC values, which are the minimum concentration of the compound/drug that inhibited the yeast growth, all the plates were kept at 37 °C for 48 h. Later, the minimum fungicidal concentration (MFC) was estimated by further growing the cells from each well at 37 °C for 24 h on Sabouraud dextrose agar (SDA; Merck, Darmstadt, Germany). Again, the lowest concentration with less than five colonies on the agar plate was recorded as the MFC.

### 2.5. Effect on Cell Viability and Count

The candidacidal phenomenon of CC-AgNPs was quantified using the count and viability kit provided by Muse^TM^. Briefly, yeast cells were grown for 8–10 h in Sabouraud dextrose broth (SDB) (Merck (Pty) Ltd., Johannesburg, South Africa) followed by centrifugation (3000 rpm, 5 min) and resuspension in fresh growth media. The yeast cells were adjusted to a density of 1 × 10^6^ CFU/mL and subjected to various strengths of test NPs (0.5 MIC, MIC, and 2 MIC) for 4 h. Afterward, the yeast cells were washed and mixed with a Muse^TM^ kit reagent (20 µL of yeast cells + 380 µL reagent), then incubated for 5 min at room temperature. The viability and cell count were estimated using a Muse^TM^ cell analyzer. The experiment included a negative and positive control (H_2_O_2_, 10 mM; Merck, Darmstadt, Germany).

### 2.6. Effect on Cell Cycle

The impact of NPs on the yeast cell cycle was investigated using a Muse™ cell cycle kit following the steps given by the manufacturer. Briefly, the cells were propagated for 8–10 h in a fresh medium (SDB) and then centrifuged at 3000 rpm for 4 min. Then, the cells were re-suspended in a fresh medium and the turbidity was adjusted to 1 × 10^6^ CFU/mL. Later, the cells were subjected to various strengths of CC-AgNPs (0.5 MIC, MIC, and 2 MIC) for 4 h. In post-incubation, the cells were washed and fixed in chilled 70% ethyl alcohol (Sigma Aldrich Co., St. Louis, MO, USA), mixed with cell cycle reagent in equal proportions, and incubated in the dark for 30 min. The experiment included both negative and positive control.

### 2.7. Effect on Mitochondrial Membrane Potential

The impact of test NPs on the mitochondrial membrane potential of *C. auris* was measured using a JC-10 assay kit (Abcam, Cambridge, UK). The experiment was done using the steps given by the manufacturer. The cells (mid-log phase) were subjected to various concentrations of test NPs (0.5 MIC, MIC, and 2 MIC) for 4 h. They were subjected to protoplast preparation, as described previously by Lone et al. [50]. Then, 90 μL of *C. auris* protoplasts were mixed with 50 µL JC-10 dye and distributed in different wells of a 96-well microtiter plate (clear bottom-black walled; Thermo Fisher Scientific, Dreieich, Germany) for 1 h in the dark. After that, 50 μL of buffer-B was added to the plate and centrifuged for 2 min at 800 rpm. The readings were captured at Ex/Em = 490/530 nm (X) and 540/590 nm (Y) in microplate readers (Molecular Devices, San Jose, CA, USA). The variation was measured in terms of the Y/X ratio. A decreased ratio confirmed the depolarization of the mitochondrial membrane. Moreover, the experiment included both negative and positive control.

### 2.8. Effect on DNA Fragmentation

The DNA fragmentation and condensation in *C. auris* MRL6057 due to CC-AgNPs were examined using a terminal deoxynucleotidyl transferase dUTP nick-end labeling (TUNEL) assay. The protocol used the In Situ Cell Death Detection Kit, Fluorescein (Roche Diagnostics, Mannheim, Germany), and the instructions provided by the manufacturer were followed. Briefly, *C. auris* cells were subjected to various concentrations of NPs (0.5 MIC, MIC, and 2 MIC) for 4 h and were subjected to protoplast preparation. Later, Triton X-100 (0.25%) was used to permeabilize the protoplasts, followed by incubation at 37 °C for 20 min. Later, the cells were mixed with TUNEL reagent and incubated at 37 °C for 1 h in a dark humidified box. Subsequently, samples were examined with fluorescence microscopy at Ex/Em = 495/519 nm (Carl Zeiss Microscopy, Jena, Germany). The experiment included both negative and positive control.

### 2.9. Haemolytic Activity

The cytotoxic potential of various concentrations of given NPs (0.5MIC, MIC, and 2MIC) was evaluated on horse RBCs (NHLS, Johannesburg, South Africa) and stated as percent hemolysis. The method was adopted from a method reported elsewhere [50]. One percent Triton X-100 was considered as the positive control, and sterile PBS solution was considered the negative control. The calculation for percent hemolysis was as follows:

(1) $$\%\;Hemolysis=\frac{\left[\left(A450\;of\;treated\;sample\right)-\left(A450\;of\;negative\;control\right)\right]}{\left[\left(A450\;of\;positive\;control\right)-\left(A450\;of\;negative\;control\right)\right]}\times 100$$

Experiments were repeated thrice, and a two-way ANOVA test was used for determining the statistical significance of the results. Additionally, *p*-values ≤ 0.05 were measured statistically.

## 3. Results and Discussion

**Figure 1:**
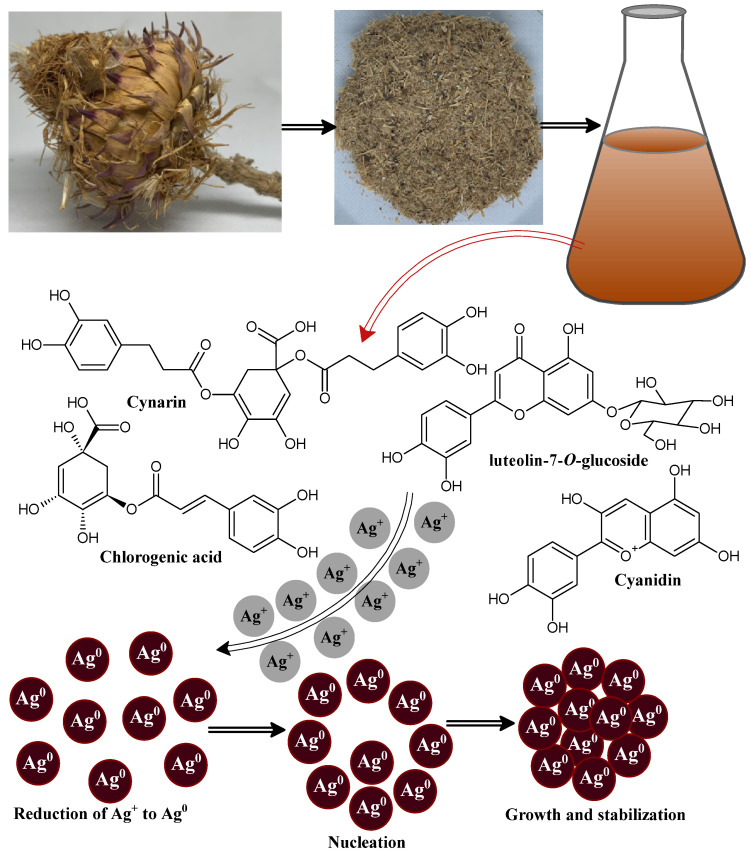
Schematic diagram showing the possible mechanism after the biosynthesis of CC-AgNPs using *Cynara cardunculus* extract.

The green and sustainable fabrication of metal nanomaterials using polyphenolic compounds has attracted huge attention because it has more advantages over the chemical route synthesis of nanomaterials [51,52]. Here, we demonstrated a simple polyphenol-capped biogenic reduction method to prepare silver nanoparticles using *Cynara cardunculus* extract as a reducing and capping/stabilizing agent. The incorporation of *Cynara cardunculus* extract in silver nitrate aqueous solution resulted in a gradual color change of the reaction mixture from pale yellow to brown and finally to a deep brown, which implied the formation of stable CC-AgNPs. The existing water-soluble polyphenolic molecules in *Cynara cardunculus* extract successfully reduced the available silver metal ion (Ag^+^) to metallic silver nanoparticles (Ag^0^) [53]. The bioreduction of metal ions to metallic nanoparticles takes place through an activation step, which involves the reduction of available silver metal ions followed by the nucleation and growth steps in which metal nanoparticles of definite shapes and sizes are formed [54]. Finally, the stabilization step takes place, in which the *Cynara cardunculus* extract metabolites similarly play the role of surface-capping agents to prevent the nanoparticles from agglomerating [54]. The proposed mechanism of CC-AgNPs formation via *Cynara cardunculus* extract is depicted in systematic Figure 1.

*Cynara cardunculus*, commonly named the artichoke plant, originated in southern Europe, and belongs to the family Asteraceae, which includes daisies and sunflowers, and is mostly cultivated as a horticultural crop in Italy [55,56,57]. The different parts of this plant possess the phytochemical composition, including (i) phenolic acid derivatives: mono and di-caffeoylquinic acid compounds [58,59] and neochlorogenic and chlorogenic acids [60,61]; (ii) flavonoids: luteolin, luteolin-7-O-glycoside, and luteolin-7-O-rutinoside [62]; (iii) sesquiterpene glycosides: cynarascolo-side A/B and cynarascoloside C; (iv) sesquiterpene lactones: cynaropicrin and grossheimin [63]; (v) triterpene saponins, including cynarasaponin E, J, C, A/H, and F/I; and (vi) the presence of amino and fatty acids was reported by Farag et al. in *Cynara cardunculus* extract, mostly concentrated in the roots, including hydroxy-octadecatrienoic acid, hydroxy-oxo-octadecatrienoic acid, tyrosyl-l-leucin, and dihydroxy-octadecatrienoic acid [64].

**Figure 2:**
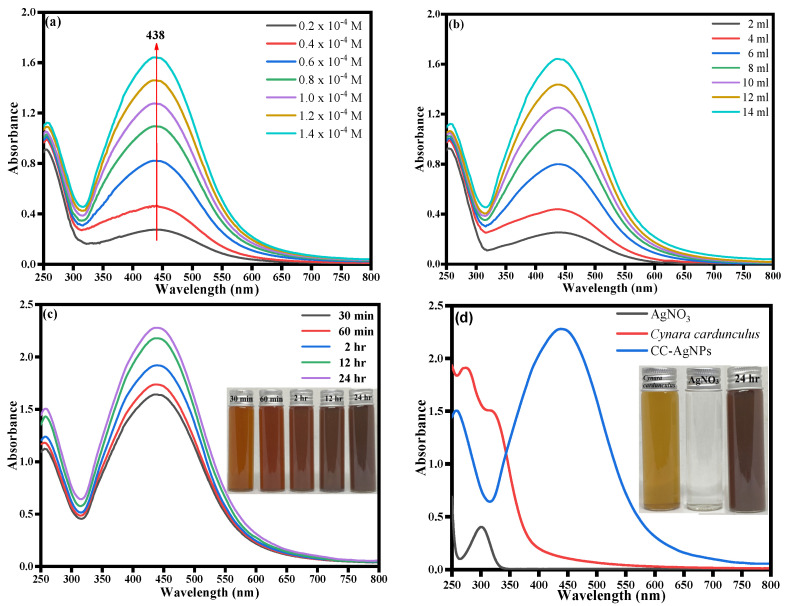
UV-vis absorption spectra of CC-AgNPs recorded as a function of different (**a**) AgNO_3_ concentrations and (**b**) plant extract concentrations; (**c**) UV-visible spectra at different times from 30 min to 360 min (inset: optical images of CC-AgNPs at different time intervals); and (**d**) UV-visible spectra of silver nitrate solution, *Cynara cardunculus* extract, and CC-AgNPs (inset: optical images of *Cynara cardunculus* extract, silver nitrate solution, and CC-AgNPs).

The UV-visible spectra were obtained as the initial analysis to record the formation of *Cynara*-*cardunculus*-extract-mediated CC-AgNPs. In general, UV-vis spectra were analyzed to infer valuable information about the shape, size, and distribution of the biosynthesized nanoparticles established using surface plasmon resonance (SPR) bands [65]. The UV-vis spectrum is important for deducing the role played by plant extract with inherited phytochemical bioactive compounds being involved in the biosynthesis of CC-AgNPs. UV-visible spectroscopy is a useful technique to determine the SPR band of the noble metal silver nanoparticles (plasmonic) due to the free electron excitation [65]. The plasmonic nanoparticles are quite distinguished from other magnetic, polymeric, and semiconductor nanoparticles because of their unique surface plasmon resonance [65]. The position of the SPR peak generally depends on the shape, state of aggregation, and particle size of the nanoparticles [65,66,67,68]. In this study, the initial physical observation of color change in colorless silver nitrate solution with the addition of *Cynara cardunculus* extract confirmed the biosynthesis of polyphenol capped AgNPs. Meanwhile, with the addition of *Cynara cardunculus* extract to colorless silver nitrate solution, the color of the reaction mixture changed from light yellow to brown within 5 to 10 min of incubation. Furthermore, the reaction mixture turned dark brown after 30 min of incubation, demonstrating the reduction of silver metal ions (Ag^+^) to silver nanoparticles (Ag^0^). The *Cynara cardunculus* extract phytochemicals acted as reducing and stabilizing agents in the biogenic synthesis of stable CC-AgNPs. The present study involved further exploration using different AgNO_3_ concentrations and different volumes of *Cynara* *cardunculus* aqueous extract, and the stability of the as-prepared CC-AgNPs was also investigated by recording the UV-visible spectra at different times from 30 min to 360 min, as shown in Figure 2a–c. Figure 2a clearly shows a sharp peak maximum at ca. 438 nm; meanwhile, with an increase in time and concentration of AgNO_3_, the increased absorption intensities were predominantly intensified. The fact of increased intensity with increased concentration of AgNO_3_ also emphasized the biogenesis of CC-AgNPs from the *Cynara cardunculus* extract increases. It is worth mentioning that the observed increase in the absorption intensity with the increase in time was due to the reduction of silver ions (Ag^+^) to elemental silver (Ag^0^). To determine the role of increased plant extract on the biosynthesis of CC-AgNPs, different plant extract concentrations ranging from 2 mL to 14 mL were explored under UV-vis spectra, as depicted in Figure 2b. The observed data revealed that with an increase in the concentration of plant extract, an increased absorbance intensity was obtained, with a maximum peak intensity at ca. 438 nm, further demonstrating the biogenesis of many spherical CC-AgNPs at higher concentrations of *Cynara cardunculus* extract. The study was further extended to establish the role of different time intervals ranging from 30 min–24 h under optimal reaction conditions, as depicted in Figure 2c. Figure 2d shows the UV-vis spectrum of the silver nitrate solution, *Cynara cardunculus* extract, and CC-AgNPs under optimal reaction conditions. Furthermore, the UV-visible spectra data revealed that the absorbance intensity of the reaction mixture increased with time, and the solution remained steady after more than 24 h of incubation, indicating that stable nanoparticle formation in the solution was successfully completed [69,70]. The optical images of silver nanoparticle formation shown in Figure 2c,d (inset) also confirmed the formation of CC-AgNPs with an increase in color intensity with incubation time from 30 min to 24 h. Subsequently, the CC-AgNPs were centrifuged, washed, dried, and calcined before being used for further studies.

**Figure 3:**
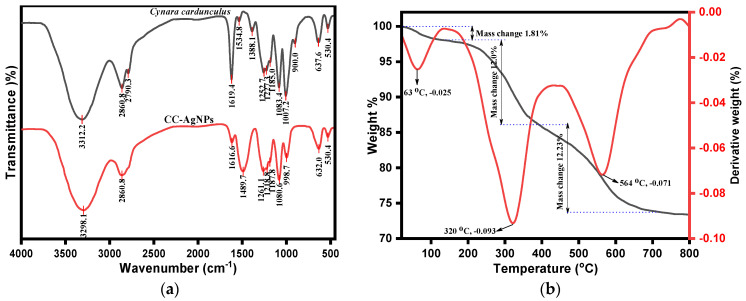
(**a**) FTIR spectra and (**b**) TGA/DTG analysis of biogenic CC-AgNPs.

The FTIR analysis was used to deduce the role of different functional groups present in the phytochemical composition of *Cynara cardunculus* extract as reducing/stabilizing agents in the biogenesis of CC-AgNPs, as depicted in Figure 3a. However, the standard peaks of CC-AgNPs were compared with *Cynara cardunculus* extract to analyze the biosynthesis of CC-AgNPs. Figure 3 reveals the presence of absorption peaks in CC-AgNPs at 3298.1 cm^−1^, 2860.8 cm^−1^, 1616.6 cm^−1^, 1489.7 cm^−1^, 1261.1 cm^−1^, 1218.8 cm^−1^, 1187.8 cm^−1^, 1080.6 cm^−1^, 998.7 cm^−1^, 632.0 cm^−1^, and 530.4 cm^−1^. However, the *Cynara cardunculus* extract possessed similar peaks as observed in standard CC-AgNPs, which demonstrated the biogenesis of CC-AgNPs upon phytochemical reduction. The peak intensity band that appeared at approximately 3300 cm^−1^, i.e., 3312.2 cm^−1^ and 3298.1 cm^−1^, were attributed to the presence of polyphenolic or polysaccharide –OH stretching vibrations. However, it was reported that the enol forms of such polyphenols or polysaccharides are reduced to quinone in extract mixture and are usually interpreted as a peak shift of –OH groups toward a higher frequency ranging between 3400 cm^−1^ and 3456 cm^−1^ [71]. A protruding peak intensity appeared at 2860.8 cm^−1^, which was assigned to C–H typical stretching vibrations from the CH_2_ groups of aliphatic compounds and are believed to have occurred after the reduction of AgNO_3_ [72]. The existence of sharp peak intensity in the range between 2820 cm^−1^ and 2760 cm^−1^, i.e., 2790.3 cm^−1^, in the *Cynara cardunculus* extract was attributed to the presence of N-CH_3_ and C-H stretching vibrations corresponding to methyl-amino substituted groups [73]. In addition, the appeared peaks at 1619.4 cm^−1^ and 1616.6 cm^−1^ lying between 1650 cm^−1^ and 1600 cm^−1^ corresponded to stretching vibrations due to conjugated ketones and aromatic ring stretching (-C=C-C) vibrations. The presence of peaks between 1550 cm^−1^ and 1400 cm^−1^, for example, 1534.8 cm^−1^ and 1489.7 cm^−1^, is believed to be due to the N-O stretching vibration of aromatic nitro compounds. The existence of an intensity peak in the range between 1410 cm^−1^ and 1310 cm^−1^, such as the 1388.1 cm^−1^ peak, in the *Cynara cardunculus* extract was assigned to –OH bending in phenolic and tertiary alcoholic groups. Similarly, the peak at 1252.7 cm^−1^ in *Cynara cardunculus* extract and the peak at 1261.1 cm^−1^ in CC-AgNPs were due to the presence of C-N stretching vibrations of primary aromatic amines. In addition, the occurrence of sharp peak intensities at 1083.4 cm^−1^ and 1080.6 cm^−1^ were the corresponding C-N stretching vibration bands of aliphatic amines, in the *Cynara cardunculus* extract and CC-AgNPs, respectively. Moreover, the peaks between 1320 cm^−1^ and 1210 cm^−1^, viz., 1227.3 cm^−1^ of the *Cynara cardunculus* extract and 1218.8 cm^−1^ of the CC-AgNPs, were attributed to C-O stretching vibrations. Furthermore, the peaks between 1190 cm^−1^ and 1130 cm^−1^, i.e., 1185.0 cm^−1^ of the *Cynara cardunculus* L. extract and 1187.8 cm^−1^ of the CC-AgNPs, signified the presence of C-N stretching vibrations of secondary amines. The observed sharp intensity peak in the range 1055 cm^−1^–1000 cm^−1^, i.e., 1007.2 cm^−1^ of the *Cynara cardunculus* extract, was due to the corresponding cyclohexane ring vibrations. The observed peak intensities at relatively lower frequencies between 850 cm^−1^ to 1000 cm^−1^, i.e., 900.0 cm^−1^ of the *Cynara cardunculus* extract and 998.7 cm^-1^ of the CC-AgNPs, were attributed to the hydrogen-bonded OH out-of-plane bending vibration modes. Meanwhile, the observed peaks between 660 cm^−1^ and 630 cm^−1^, such as 637.6 cm^−1^ of the *Cynara cardunculus* L. extract and 632.0 cm^−1^ of the CC-AgNPs, were believed to occur due to the presence of C-S thio-substituted compounds. The important observed peak intensity in both *Cynara cardunculus L.* extract and CC-AgNPs at 530.4 cm^−1^ was caused by the reduction of Ag^+^ to Ag^0^ in the biosynthesis of CC-AgNPs from *Cynara cardunculus* aqueous extract. In conclusion, the presence of polyphenolic vibrations, along with other functional group stretching vibrations, of *Cynara cardunculus* extract demonstrated their role as reducing and stabilizing agents in the biosynthesis of CC-AgNPs.

The phytochemicals play essential roles as stabilizing and capping agents in the biogenesis of nanoparticles. Thus, the weight loss and thermal stability of such were investigated, which depended upon the adsorption of phytochemicals onto the surface of biogenic CC-AgNPs. Although the thermogravimetric analysis was operated at a heating rate of 10 °C/min under a nitrogen atmosphere, we inferred the weight loss in a stepwise manner for biogenic CC-AgNPs, as depicted in Figure 3b. The overall weight loss in this study of thermal decomposition associated with CC-AgNPs from *Cynara cardunculus* extract was approximately 26.64% by weight. This observed weight loss in the first region between 0–220 °C was 1.81% by weight occurred after the loss of the adsorbed surrounding moisture and volatile residues of phytochemicals onto the surface of CC-AgNPs. Furthermore, the observed TGA peak at 63 °C for the derivative weight (by %) of −0.025 corresponded to the degradation of volatile phytocompounds onto the surface of the biosynthesized CC-AgNPs. The second weight loss between 150–300 °C was 12.6% by weight, which was observed after the thermal degradation of heterocyclic volatile phytochemical compounds that thermally decomposed onto the adsorbed surface of CC-AgNPs. The third region of thermal decomposition after 300 °C, with the DTG peak at 320 °C for the derivative weight (by %) of −0.093 and the DTG peak at 564 °C for the derivative weight (by %) of −0.071 were attributed to the thermal decomposition with a weight loss (by % weight) of about 12.23%, which was believed to occur after the thermal decomposition of phytochemical constituents, including polyphenolic compounds, flavonoids, and polysaccharides. These phytocompounds have an important appealing role in the capping and stabilization of the biosynthesized surface morphology of CC-AgNPs. The overall results of the TGA/DTG demonstrated the thermally stable biosynthesis of CC-AgNPs from *Cynara cardunculus* extract.

**Figure 4:**
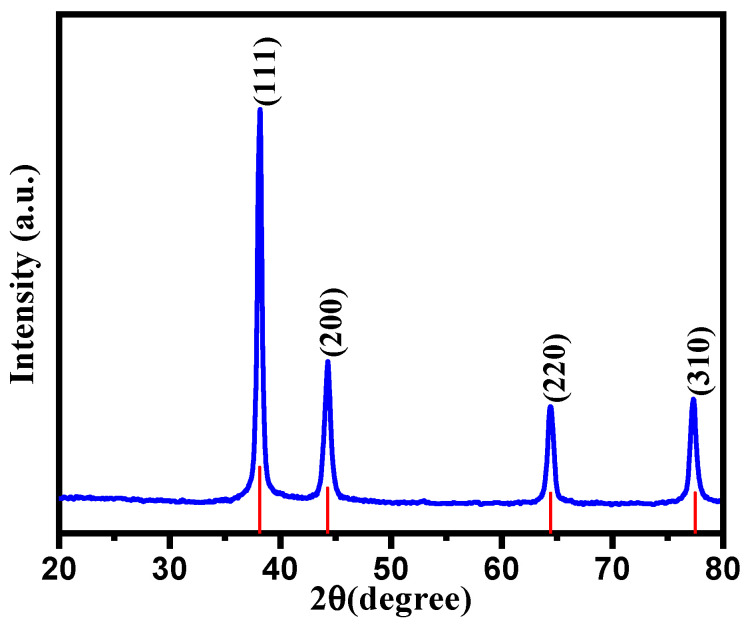
XRD spectrum of CC-AgNPs from the *Cynara cardunculus* extract.

The as-synthesized CC-AgNPs were subjected to XRD analysis to deduce the crystalline nature of the particles. The XRD pattern of the green-synthesized CC-AgNPs from *Cynara cardunculus* extract was obtained and is depicted in Figure 4. It is clear from Figure 4 that the diffraction peaks observed at 2θ angles of 38.16° (111), 44.40° (200), 64.58° (220) and 77.38° (311) corresponded to the face-centered cubic (fcc) structure of metallic silver. Moreover, the obtained XRD results were a good approximation to the JCPDS Card NO. 04-0783 results [74]. However, the presence of a prominent peak at 38.16° was due to the crystalline Ag, showing that the biosynthesized CC-AgNPs were encompassed with crystalline Ag lattice sites. The average particle size found via XRD analysis was calculated using the Scherrer equation (d = Kλ/βcosθ), where d is the crystallite size, K represents the Scherer constant equal to 0.9, λ is the wavelength of the X-ray source (typically 1.5406 Å), β is the FWHM in radians, and θ is the peak position (Bragg angle), as tabulated in Table 1. The average crystalline size was found to be 17.26 nm by using the Scherrer calculation; meanwhile, similar numerical values of crystalline size were found in the SEM analysis, as discussed in another section in detail. No additional reflection other than Ag-lattices was observed, demonstrating the purity of the biosynthesized CC-AgNPs from the *Cynara cardunculus* extract.

**Figure 5:**
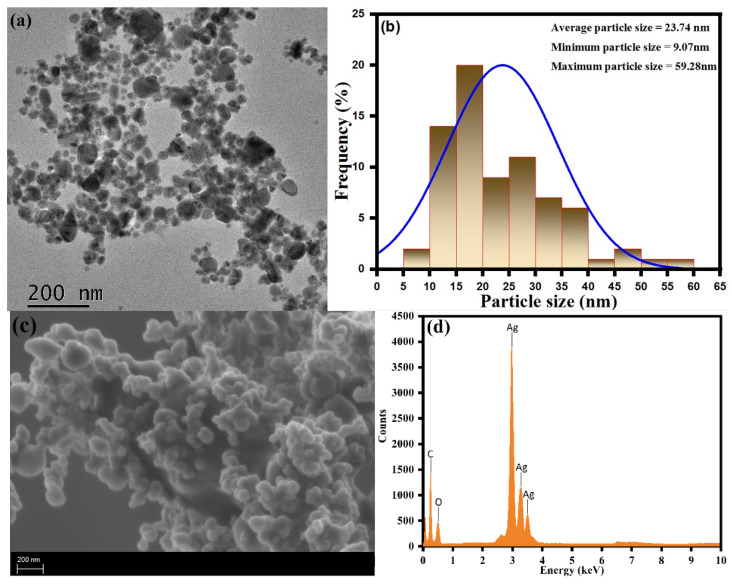
(**a**) TEM, (**b**) particle size histogram, (**c**) SEM, and (**d**) EDX spectra of the CC-AgNPs.

The transmission electron microscopy (TEM) and scanning electron microscopy–energy-dispersive X-ray spectroscopy (SEM-EDX) analysis of the as-synthesized CC-AgNPs were used to perform the study of the exterior surface morphology, structural characterization, and elemental composition, as shown in Figure 5. The TEM analysis of the green-synthesized CC-AgNPs from *Cynara cardunculus aqueous* extract showed them to be well defined with a homogenous distribution of nearly spherical nanoparticles with an average particle size ranging between 14 to 43 nm. The larger particle sizes were observed due to the formation of agglomerates of the small particles. The average (23.74 nm), minimum (9.07 nm), and maximum (59.28 nm) particle sizes of the as-prepared CC-AgNPs were found from the particle size histogram using ImageJ software, as shown in Figure 5b. During the SEM analysis, the surface morphology showed agglomeration of spherical CC-AgNPs, as shown in Figure 5c. Besides the SEM analysis, EDX analysis was performed for the elemental analysis and purity of the as-prepared CC-AgNPs, as shown in Figure 5d. The observed intensity ranges emphasized the strong spectral signals corresponding to the silver region (Ag). The observed sharp intensity signal at 3 KeV was found due to the adsorption of the metallic silver region and emphasized the presence of biosynthesized nanocrystallites of CC-AgNPs. The other signals found in the 0–0.5 KeV range were attributed to the presence of adsorbed oxygen and carbon atoms. The overall results of surface morphology and elemental analysis indicated that the biosynthesized CC-AgNPs using *Cynara cardunculus aqueous* extract were of high purity.

**Figure 6:**
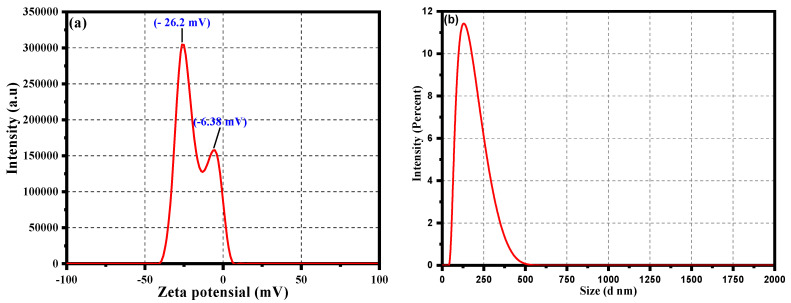
(**a**) Zeta potential and (**b**) particle size distribution analysis of the CC-AgNPs.

A zeta potential analysis was undertaken to analyze the stability of the biosynthesized CC-AgNPs from *Cynara cardunculus* extract in their colloidal state. The results obtained using zeta potential analysis are depicted in Figure 6a. Values of zeta = −26.2 mV and −6.38 mV was observed, showing a negative charge on the surface of the CC-AgNPs, further emphasizing their stability in the colloidal state and the role of phytochemicals as surface-capping agents. The observed negative values of the zeta potential were due to from the absorbed phytochemicals with negative surface charge onto the surface, possibly because of the presence of functional groups, such as OH-, CO-, and COO-. However, the detailed mechanism and possibility of occurrence of such functional groups are discussed in another section related to characterization using FTIR analysis. In general, the presence of the negative surface charge on the surface of CC-AgNPs resulted in preventing the aggregation and stabilizing the CC-AgNPs upon electrostatic repulsion among the negative charges. Moreover, the size distribution of the as-synthesized CC-AgNPs was analyzed using dynamic light scattering (DLS) analysis, as shown in Figure 6b. The DLS results of the CC-AgNPs shown in Figure 6b revealed that the average size of the particles in the optimal condition was 127 nm with a polydispersity (PDI) of 0.515. The bigger hydrodynamic diameter shown in the DLS results as compared with the TEM and XRD results was because of the presence of the capping agents and some agglomerated CC-AgNPs in the reaction mixture.

### 3.1. Anti-Candida Activity of CC-AgNPs

The CC-AgNPs were found to be active against *C. auris* MRL6057, and the MIC values were reported as 50.0 µg/mL, whereas the MFC was found to be 100.0 µg/mL. In comparison, the MIC and MFC values of AmB against *C. auris* MRL6057 were found to be 4.0 and 8.0 µg/mL, respectively. According to published MIC breakpoints for *C. auris* [75], the clinical strains of *C. auris* with MIC ≥2 µg/mL are considered resistant to AmB. Therefore, *C. auris* MRL6057 was deemed to be resistant to AmB.

*C. auris* displays high resistance to commonly used drugs [76]. In vitro examinations demonstrated the reduced susceptibility of ≥90% of *C. auris* isolates to fluconazole [77]. In comparison, 3–73% and 13–35% of clinical isolates of *C. auris* were found to be resistant to voriconazole and AmB, respectively [78,79]. Furthermore, in the USA, ≥99% of these isolates were reported to be less susceptible to fluconazole, around two-thirds were resistant to AmB, and approximately 4% were found to be resistant to echinocandins class antifungals [80]. The global emergence of pan-resistant strains of *C. auris* and their ability to persist in healthcare settings has redrawn the attention of researchers and healthcare experts to this pathogenic yeast [79].

Researchers investigated the candidacidal potential of AgNPs against *C. albicans*, and AgNPs were found to be potential inhibitors of growth and viability, both alone and in combination; for instance, a low strength of 1.8 mg/mL AgNPs in combination to cationic carboxilane inhibited the growth of *C. albicans* [81]. Additionally, L-3,4-dihydroxyphenylalanine capped with AgNPs showed fungicidal activity at a concentration of 31.2–62.5 μg/mL [82]. These findings support our investigation and that AgNPs can be a potential candidate for drug development against *C. auris*. Therefore, further research that analyzed the in-depth mechanism of antifungal action of CC-AgNPs was required.

### 3.2. CC-AgNPs Impede Cell Count and Viability in C. auris

**Figure 7:**
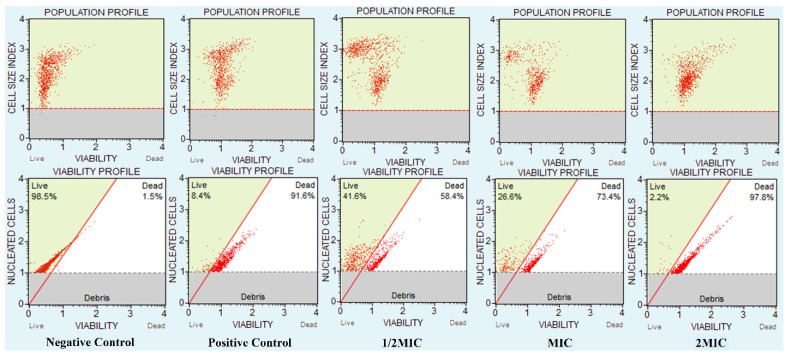
CC-AgNPs affected *C. auris* cell numbers and viability. The figure shows the *C. auris* viability and population profile. Negative control: unexposed *C. auris* cells; positive control: H_2_O_2_ exposed cells; *C. auris* exposed to various MIC values of the test NPs.

A susceptibility assay of *C. auris* was performed against CC-AgNPs to measure the growth and viability of these cells (Figure 7). The unexposed cells had 98.5% live cells, whereas, after exposure to H_2_O_2_, the percentage of live cells was 8.4%. Moreover, the reduction in the number of live cells was dependent on the concentration of the NPs. Therefore, the percentage of live cells decreased with increasing concentration of NPs, where 41.6%, 26.6%, and 2.2% live cells were recorded at values of 0.5MIC, MIC, and 2MIC, respectively. The results confirmed that these NPs entirely stopped the growth and survival of *C. auris*, and thus, corroborated the anti-*Candida* potency of AgNPs.

The antimicrobial activity of AgNPs was well studied by various researchers [81,82]. The current findings were in accord with the results obtained by Wani and co-workers 2013, where they showed the potent anti-*Candida* effect of metallic NPs [83]. The antifungal activity of metallic NPs is attributed to their ability to disrupt the membrane porosity and induce cellular damage, ROS production, damage of nucleic acid, and disruption of important biological enzymes [84].

### 3.3. CC-AgNPs Arrested the Cell Cycle in C. auris

The CC-AgNPs may result in the induction of cellular apoptosis in *C. auris,* and thus, the potency of these NPs on the cell cycle was investigated. Consequently, the number of cells dispersed in various phases of the cell cycle must be different from that present in the healthy cells, representing cell cycle blockage. Hence, the change in DNA content was evaluated quantitatively with the help of fluorescence generated using DNA tagged with PI throughout the cellular growth.

**Figure 8:**
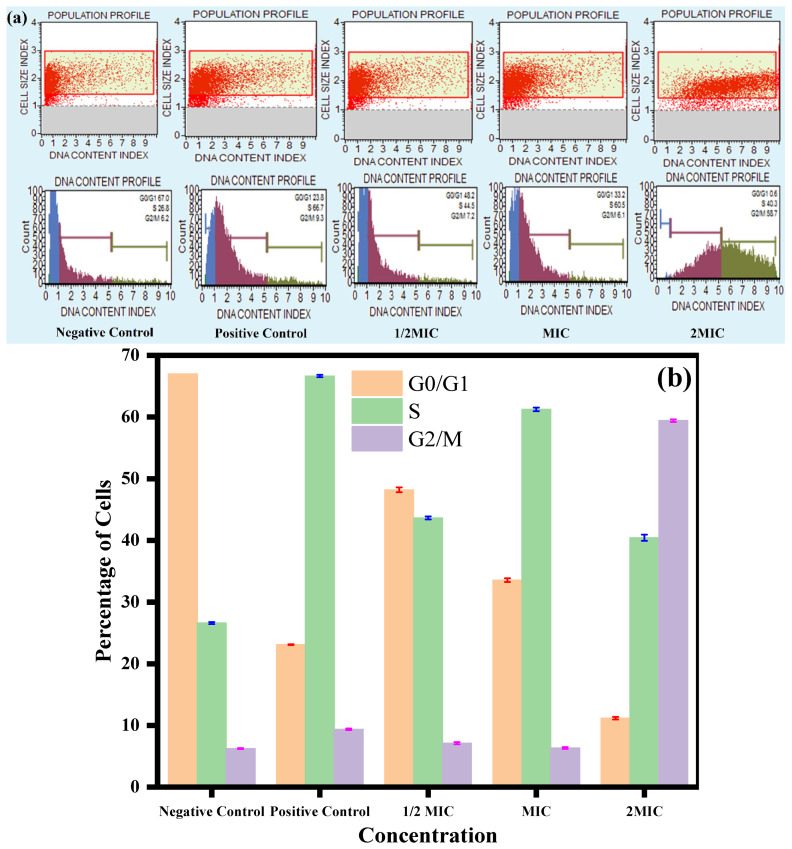
Cell cycle analysis of *C. auris*. (**a**) Effect of CC-AgNPs at various concentrations on cell cycle progression in *C. auris*. (**b**) Representative histograms of the *C. auris* cell cycle at various CC-AgNP concentrations. Positive controls were cells treated with H_2_O_2_ (10 mM) and negative controls were untreated cells.

The results are summarized in Table 2, where the unexposed experiment had the maximum number of cells in the G0/G1 phase, followed by the S phase and G2/M phase, whereas, the cells of the positive control were mostly accumulated in the S phase, followed by the G0/G1 and G2/M phase. However, after exposure to the CC-AgNPs, the cell cycle was blocked at two different phases: the S phase and G2/M phase (Figure 8a,b). At 0.5 MIC and MIC, the cells were arrested in the S phase of the cell cycle, with the distribution percentages of 44.5% and 60.2%, respectively. Furthermore, at 2MIC, the cells were arrested in the G2/M phase (58.7%). Altogether, the present findings discovered that the CC-AgNPs at a lower concentration arrested the cells in the S phase, whereas, at a higher concentration, the G2/M phase is blocked, and therefore, had a conspicuous role in blocking cell cycle advancement in *Candida*.

These findings were in good agreement with previous results where researchers showed the inhibitory effect of various compounds on the cell cycle of *Candida* species. For instance, clioquinol, crambescidin-816, and crambescidin-089 were found to block the G2/M phase in *Candida* and Saccharomyces cerevisiae [85,86,87]. Furthermore, the damaged cell cycle alters the cellular morphology, which increases the chance of the host’s immune system recognizing the yeast cells [88]. Thus, CC-AgNPs can impede the cell cycle in *C. auris* and boost its identification by the host’s immune system.

### 3.4. CC-AgNPs Depolarized the Mitochondrial Membrane Potential in C. auris

**Figure 9:**
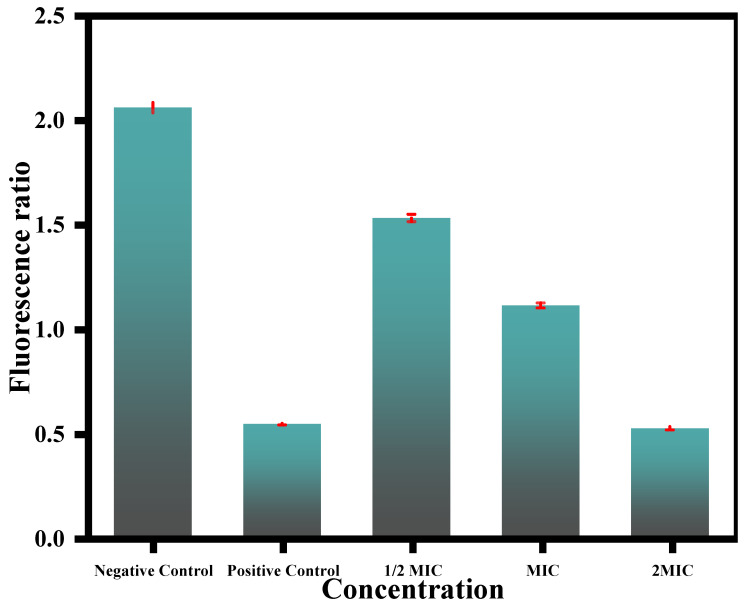
Effect of CC-AgNPs at varying concentrations on mitochondrial membrane depolarization in *C. auris* cells. Positive and negative controls were represented by cells treated with 10 mM H_2_O_2_ and untreated cells, respectively.

The mitochondrial membrane potential is one of the primary steps in fungal apoptosis owing to the mitochondria’s crucial role in cell survival. The results obtained in this study revealed the potential of the test CC-AgNPs to cause mitochondrial membrane disintegration in *C. auris* (Figure 9). Mitochondrial membrane disruption results in pore formation, which leads to the depolarization and movement of different apoptotic factors. Mitochondrial depolarization was also related to the cytochrome c release and was often observed during early apoptosis. The test CC-AgNPs caused mitochondrial toxicity induction, which was followed by the loss of membrane potential, oxidative phosphorylation inhibition, and changes in calcium sequestration [89]. Other studies reported the potential of silver nanoparticles to depolarize mitochondrial membranes and cause apoptosis in *C. albicans,* which was related to an increase in hydroxyl radicals [90]. A study by Zhu and co-workers reported the impact of iron nanoparticles in causing apoptosis in human umbilical endothelial cells by causing membrane depolarization [90]. These studies further supported our claims and are congruent with our findings that metal nanoparticles can cause mitochondrial membrane depolarization and with our conclusions that metal nanoparticles can cause mitochondrial membrane depolarization and apoptosis in fungal cells. However, to further verify these claims, more studies involving other metal nanoparticles, including mono-, bi-, and/or tri- metallic nanoparticles, are needed to compare their effects in different fungal cells in terms of causing apoptosis related to mitochondrial membrane depolarization.

### 3.5. The CC-AgNPs Elicited DNA Fragmentation in C. auris

**Figure 10:**
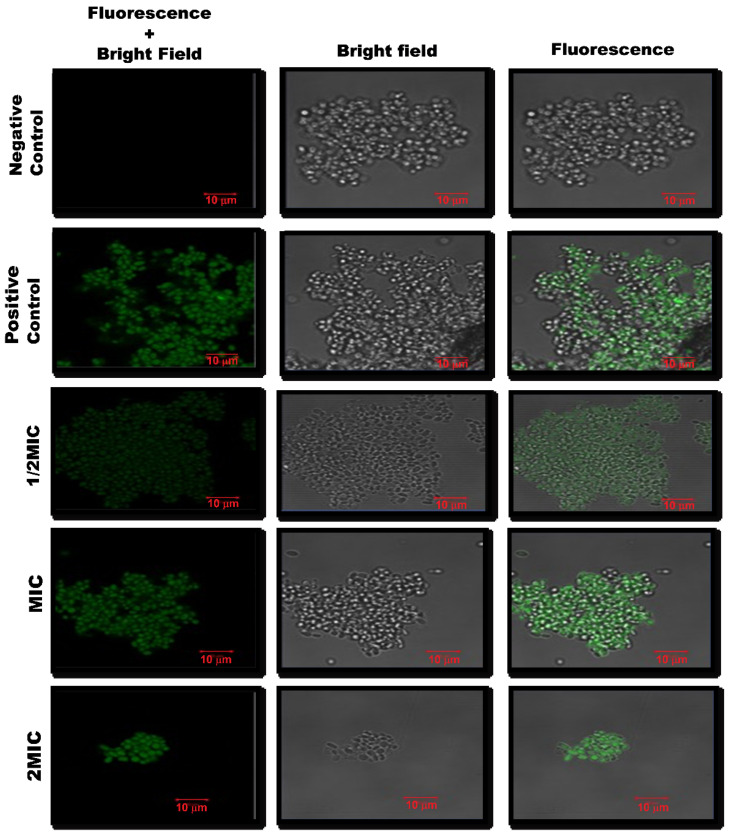
Confocal scanning fluorescence images of *C. auris* when treated with different concentrations of CC-AgNPs. Untreated cells were the negative control, whereas cells treated with H_2_O_2_ (10 mM) were the positive control.

The microscopic analysis for the TUNEL assay results revealed that at higher concentrations of CC-AgNPs, green solid fluorescence suggested DNA breakage, as observed for cells treated with 10 mM H_2_O_2_ (Figure 10). Therefore, these results demonstrated the potential of the test NPs to cause DNA fragmentation in *C. auris*. At a lower concentration (25 μg/mL), the degree of DNA fragmentation was not much, which was depicted by fewer TUNEL-positive yeast cells. However, with increasing concentration (50–100 μg/mL), the degree of DNA damage also increased, which was reflected in the higher number of TUNEL-positive yeast cells.

DNA fragmentation is one of the significant markers related to morphological changes to identify the late apoptosis in yeast cells. The DNA fragmentation can be visualized using a TUNEL assay, which labels the free 3′-OH termini with modified nucleotides catalyzed by terminal deoxynucleotidyl transferase. The TUNEL assay was observed as the most dependable method to study late apoptosis [91]. The test nanoparticles’ cell cycle arrest and DNA fragmentation suggested that CC-AgNPs can damage nucleic acids in *C. auris* and other pathogenic yeasts. It was also predicted that these nanoparticles, besides damaging nucleic acids in *C. auris*, can also damage antioxidant enzymes and cause lipid peroxidation in fungal cells. Overall, the results from this study suggested that CC-AgNPs led to nucleic acid fragmentation and mitochondrial membrane depolarization, which are the characteristic markers of apoptosis, thus, validating the idea that AgNPs induce late apoptosis in yeast cells and have dual antifungal action modes, including membrane disruption. The overall result suggested that CC-AgNPs led to mitochondrial membrane depolarization and DNA fragmentation, which are crucial apoptosis characteristics.

**Figure 11:**
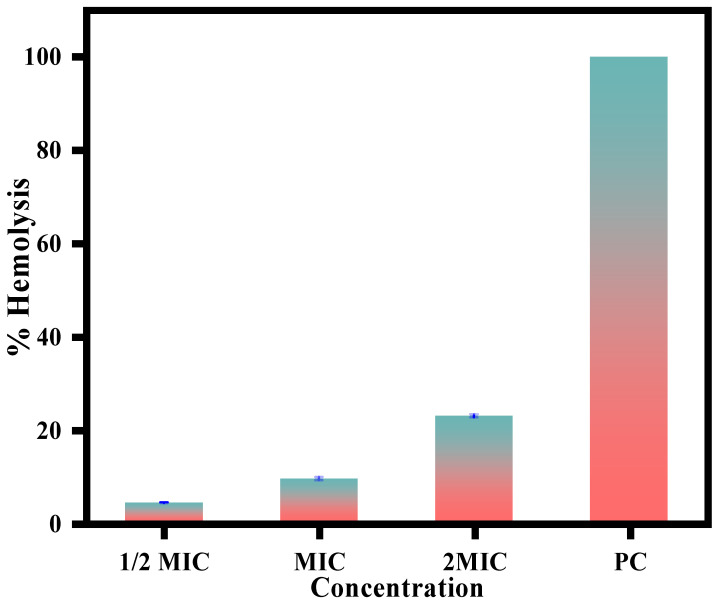
Hemolytic activity of CC-AgNPs using horse erythrocytes showed no lysis in untreated cells, whereas 100% hemolysis was caused by Triton X, which was the positive control.

The CC-AgNPs in this study showed potent antifungal activity with a dual antifungal mode of action by causing cell cycle arrest and cellular apoptosis. However, to further escalate these nanoparticles to the next steps of drug development, it is essential to check their toxicity on host cells. To this end, the CC-AgNPs were tested for hemolytic activity against horse blood cells. The results obtained in this study revealed only 3–7% cell lysis when treated with CC-AgNPs at ½ half of MIC and MIC respectively. Figure 11, thus confirming that CC-AgNPs are safe for in vivo animal experiments. Even at higher concentrations (2MIC), only 13% hemolysis was observed; however, this concentration is not considered safe for testing in animal models. Furthermore, our results also reported no lysis observed in untreated control cells, whereas 100% cell lysis was observed with Triton X, which served as the positive control.

## 4. Conclusions

In this work, chemically stable silver nanoparticles (CC-AgNPs) were prepared via a phytochemically induced synthesis process using *Cynara cardunculus* extract as a reducing and capping agent. The present work was facile, cost-effective, and ecofriendly and did not require any solvent except water, which made this process highly advantageous. The formation of CC-AgNPs was confirmed by various microscopic and spectroscopic techniques before utilizing them for antifungal activities against *C. auris*. The experimental conditions for the preparation of CC-AgNPs were optimized via a surface plasmon (SPR) peak at 438 nm using UV-visible spectroscopy. Furthermore, the CC-AgNPs directly inhibited the cell cycle and arrested cells in the G2/M phase and could be a potential lead for antifungal drug development. Our results demonstrated that the as-prepared silver nanoparticles had good antifungal performance against *C. auris* and could be further explored for exceptional and enhanced biomedical applications.

## Figures and Tables

**Table 1:** Lattice parameters and crystalline sizes of CC-AgNPs from the XRD patterns.

2θ (°)	FWHM	Miller Indices (hkl)	d_hkl_ d-Spacing (Å)	Crystal Size d (nm)	d (Average)
38.13	0.4353	(111)	2.358	19.30	17.26 nm
44.27	0.6005	(200)	2.044	14.28	
64.42	0.5394	(220)	1.445	17.40	
77.312	0.5636	(310)	1.233	18.04	

**Table 2:** Cell cycle in *C. auris*.

Experiment	Phases of Cell Cycle	Cell Percentage (%)
Negative control	G0/G1	67
	S	26.8
	G2/M	6.2
Positive control	G0/G1	23.8
	S	66.7
	G2/M	9.3
0.5MIC (25 µg/mL)	G0/G1	48.2
	S	44.5
	G2/M	7.2
MIC (50 µg/mL)	G0/G1	33.2
	S	60.2
	G2/M	6.1
2MIC (100 µg/mL)	G0/G1	0.6
	S	40.3
	G2/M	58.7

## Data Availability

All data created is provided in this study.
